# Optimization, Characterization, and Comparison of Two Luciferase-Expressing Mouse Glioblastoma Models

**DOI:** 10.3390/cancers16111997

**Published:** 2024-05-24

**Authors:** Louis T. Rodgers, Julia A. Schulz Pauly, Bryan J. Maloney, Anika M. S. Hartz, Björn Bauer

**Affiliations:** 1 Department of Pharmaceutical Sciences, College of Pharmacy, University of Kentucky, Lexington, KY 40536, USA; 2 Sanders-Brown Center on Aging, University of Kentucky, Lexington, KY 40536, USA; 3 Department of Pharmacology and Nutritional Sciences, College of Medicine, University of Kentucky, Lexington, KY 40536, USA

**Keywords:** glioblastoma, luciferase, bioluminescence imaging, preclinical, GL261 Red-FLuc, TRP-mCherry-FLuc, artificial intelligence

## Abstract

**Simple Summary:**

Inconsistent engraftment of luciferase-expressing tumors, especially GL261 Red-FLuc, has been reported in the recent literature. However, techniques to improve tumor take have not been described. Our study aimed to optimize two luciferase-expressing mouse glioblastoma models, GL261 Red-FLuc and TRP-mCherry-FLuc, which revealed differences in tumor development and characteristics. While the features of each tumor were distinct, with GL261 Red-FLuc tumors showing high mitotic activity and vascularization and TRP-mCherry-FLuc tumors displaying necrosis and invasiveness, each exhibited features reminiscent of patients with glioblastoma. Furthermore, we developed a method for high-throughput sample analysis by quantifying the luciferase-positive tumor volume using artificial intelligence. Our findings provide valuable insights for researchers using similar models, emphasizing the need to consider tumor engraftment for robust preclinical research.

**Abstract:**

Glioblastoma (GBM) is the most aggressive brain cancer. To model GBM in research, orthotopic brain tumor models, including syngeneic models like GL261 and genetically engineered mouse models like TRP, are used. In longitudinal studies, tumor growth and the treatment response are typically tracked with in vivo imaging, including bioluminescence imaging (BLI), which is quick, cost-effective, and easily quantifiable. However, BLI requires luciferase-tagged cells, and recent studies indicate that the luciferase gene can elicit an immune response, leading to tumor rejection and experimental variation. We sought to optimize the engraftment of two luciferase-expressing GBM models, GL261 Red-FLuc and TRP-mCherry-FLuc, showing differences in tumor take, with GL261 Red-FLuc cells requiring immunocompromised mice for 100% engraftment. Immunohistochemistry and MRI revealed distinct tumor characteristics: GL261 Red-FLuc tumors were well-demarcated with densely packed cells, high mitotic activity, and vascularization. In contrast, TRP-mCherry-FLuc tumors were large, invasive, and necrotic, with perivascular invasion. Quantifying the tumor volume using the HALO^®^ AI analysis platform yielded results comparable to manual measurements, providing a standardized and efficient approach for the reliable, high-throughput analysis of luciferase-expressing tumors. Our study highlights the importance of considering tumor engraftment when using luciferase-expressing GBM models, providing insights for preclinical research design.

## 1. Introduction

Glioblastoma (GBM) is one of the most aggressive forms of brain cancer, with a 5-year survival rate of only 5.5% [1]. Despite the use of the standard of care (resection, radiation, and chemotherapy with the alkylating agent temozolomide), recurrence is nearly inevitable due to the invasion of remnant GBM cells into the surrounding brain parenchyma [2,3].

In pursuing effective treatments for improving patient survival, researchers use preclinical mouse GBM models to study disease characteristics and test novel therapies. These models are broadly divided into four categories: cell-line xenografts, patient-derived xenografts, syngeneic mouse models, and genetically engineered mouse models (GEMMs) [4]. Tumors of GEMMs can be dissociated for subsequent implantation to create models known as homografts [5]. Among these, syngeneic models and homografts allow the implantation of GBM cells of murine origin into mice of a similar genetic background, resulting in tumors with highly consistent growth rates and survival. Furthermore, the parental GEMM tumors develop de novo and are derived from specific mutations shared with human GBM tumors. Examples of syngeneic models and GEMMs include mouse glioma 261 (GL261) and TRP murine GBM, respectively.

GL261 is a widely used syngeneic GBM model established through intracranial implantation of carcinogenic 3-methylcholanthrene pellets, followed by tumor harvesting [6]. Subsequent tumor maintenance techniques involve serial fragment transplantation into both the C57BL/6 mouse brain and flank [7]. Over time, in vitro cultures were established, enabling long-term propagation and widespread use as a preclinical GBM model. GL261 tumors share many pathological and molecular features with human GBM, including anaplasia, pleomorphic cells with atypical nuclei, hypoxia, angiogenesis, and an increased mitotic rate [8]. Furthermore, GL261 tumors are radio- and chemo-sensitive and harbor mutations in tumor suppressor protein 53 (p53) and Kirsten rat sarcoma viral oncogene (KRAS) [9,10,11,12]. The TRP model was derived from a GEMM harboring an inactive retinoblastoma protein (RB), constitutively active KRAS, and phosphatase and tensin homolog (PTEN) deletion, which results in the activation of the receptor tyrosine kinase (RTK)/phosphoinositide 3-kinase (PI3K) pathway and p53 missense mutations [13]. Notably, RB, RTK/PI3K and p53 pathway dysregulation is present in about 78%, 88%, and 87% of GBM tumors, respectively, with 74% harboring alterations in all three pathways [14]. These mutations lead to tumors with pathological features resembling patients’ GBM samples, including pseudopalisading necrosis, vessel co-option, and invasion [13]. These features collectively make GL261 and TRP valuable models for preclinical research.

When using preclinical intracranial GBM models, monitoring the treatment response is typically limited to traditional analyses, such as histopathology or MRI, which have limitations. Histopathology is a labor-intensive process that requires sacrificing animals, which limits downstream endpoint analyses. Contrast-enhanced MRI involves time-consuming animal preparation with tail vein catheter placement and variable scan times and sequences. The nature of these procedures does not make them feasible for high-throughput studies.

To avoid these limitations, cancer cell lines are often genetically modified to express reporter genes, such as the firefly luciferase enzyme. Luciferase expression allows for in vivo bioluminescent imaging of tumors in live animals, which yields real-time quantitation of the relative tumor size and treatment response while reducing animal usage and increasing throughput [15]. The most common luciferase reporters include Luc (original), Luc2 (next-generation codon-modified), and Red-FLuc (latest generation red-shifted, highest intensity, and suitable for deep tissue imaging) [16,17]. However, cancer cells expressing reporter proteins, including luciferase, can be immunogenic, which is associated with spontaneous tumor regression [18,19]. Sanchez et al. recently demonstrated that more than half of C57BL/6 mice implanted with GL261 Red-FLuc cells rejected their tumors when injected with cell numbers ranging from 50,000 to 300,000 cells, suggesting that further characterization and optimization of luciferase-expressing tumor models is needed [20].

For the present study, we compared the tumor take and growth characteristics of luciferase-expressing GL261 Red-FLuc and TRP-mCherry-FLuc (TRP-mCF) tumors in immunocompetent (albino B6) mice. Due to the low tumor take of GL261 Red-FLuc tumors in immunocompetent mice, we switched to immunocompromised (J:NU) mice for further tumor characterization. We then characterized the histopathology and imaging features of GL261 Red-FLuc and TRP-mCF tumors in immunocompromised and immunocompetent hosts, respectively. Implantation of luciferase-expressing GL261 Red-FLuc cells into J:NU mice increased tumor take to 100% compared to 38% in albino B6 mice while maintaining the histopathological features of this tumor. In contrast, TRP-mCherry-FLuc tumor take was 100% in 4 out of the 5 injected cell numbers. We also report a method for detecting luciferase-positive tumor cells using artificial intelligence to quantify the tumor volume and a high throughput analysis of histopathology samples. These studies directly compare two luciferase-expressing mouse GBM models, emphasizing the impact of luciferase expression on tumor engraftment, a critical aspect often overlooked in preclinical model development that is largely underreported in the existing literature.

## 2. Materials and Methods

### 2.1. Cell Line and Culture Conditions

The parental GL261 cell line was purchased from the Division of Cancer Treatment and Diagnosis Tumor Repository (National Cancer Institute, NIH, Bethesda, MD, USA), and the Bioware^®^ Brite GL261 Red-FLuc cell line was purchased from PerkinElmer (BW134246; Waltham, MA, USA). Both cell lines were cultured in Dulbecco’s modified Eagle’s medium (DMEM) containing 1000 mg/L glucose, 584 mg/L L-glutamine, 3.7 g/L sodium bicarbonate (D6046; MilliporeSigma, Saint Louis, MO, USA), and 10% fetal bovine serum (FBS; 89510-186, VWR, Radnor, PA, USA). GL261 Red-FLuc medium was supplemented with 2 µg/mL puromycin (1861; BioVision, Waltham, MA, USA) for maintaining luciferase expression. TRP-mCherry-FLuc (TRP-mCF) cells were kindly provided by Dr. Shawn Hingtgen (University of North Carolina, Chapel Hill, NC, USA) and maintained in DMEM containing 4500 mg/L glucose, 584 mg/L L-glutamine, 3.7 g/L sodium bicarbonate (Corning, Corning, NY, USA), 10% heat-inactivated fetal bovine serum, and 1x penicillin-streptomycin (MP Biomedicals, Solon, OH, USA).

Cells were incubated in a Heraeus HERAcell 150 CO_2_ incubator (Thermo Fisher Scientific, Waltham, PA, USA) at 37 °C and 5% CO_2_. Cell morphology, proliferation, and confluence were assessed at 100-fold magnification with a TELAVAL 31 inverted transmitted light microscope (Zeiss, White Plains, NY, USA). Once cells were 80–90% confluent, they were treated with 0.05% trypsin-EDTA (25-053-Cl; Corning, Corning, NY, USA) in phosphate-buffered saline (PBS with 1.05 mM KH_2_PO_4_, 154 mM NaCl, 5.6 mM Na_2_HPO_4_; SH30256.01; HyClone Laboratories, Logan, UT, USA) for 3 min at 37 °C. Trypsinization was stopped with cell culture medium at twice the added 0.05% trypsin-EDTA volume. Cells were centrifuged (200× *g*, 5 min, RT) and resuspended in cell culture medium. We used a Scepter 2.0 automated cell counter to count cells (MilliporeSigma, Saint Louis, MO, USA). Cells were regularly tested for mycoplasma using either the PCR Mycoplasma Test Kit I/C (PK-CA91-1096; PromoCell GmbH, Heidelberg, DE, Germany) or the MycoStrip™ Mycoplasma Detection Kit (InvivoGen, San Diego, CA, USA).

### 2.2. Mice

All animal experiments were approved by the University of Kentucky Institutional Animal Care and Use Committee (IACUC #2018-2947; PI: Bauer). The University of Kentucky Division of Laboratory Animal Resources is an AAALAC-accredited institution, and experiments were carried out per the US Department of Agriculture Animal Welfare Act and the Guide for the Care and Use of Laboratory Animals of the National Institutes of Health.

Eight-week-old female homozygous J:NU (immunocompromised; strain number 007850) and albino B6 (B6(Cg)-*Tyr^c−2J^*/J; immunocompetent; strain number 000058) mice were purchased from Jackson Laboratory (Bar Harbor, ME, USA). Female mice were used because the GL261 cells are female-derived, and it is conventional to maintain consistency with the gender of the tumor host for syngeneic models [20,21,22,23]. Female mice were used for the TRP-mCF model to stay consistent between models. Mice were group-housed in cages connected to an EcoFlo ventilation system (Allentown Inc., Allentown, NJ, USA) in an AAALAC-accredited temperature- and humidity-controlled facility at the University of Kentucky (21–22 °C, 30–70% humidity, 14:10-h light/dark cycle). Mice received water and standard chow ad libitum (Envigo Teklad Chow 2918, Envigo, Indianapolis, IN, USA).

### 2.3. Stereotaxic Intracranial Tumor Cell Implantation

GBM cell implantation was based on previously published protocols from Carlson et al. [24] and El Meskini et al. [25]. On the day before tumor cell implantation, the heads of albino B6 mice were shaved with a cordless hair trimmer under 2% isoflurane anesthesia. On the morning of the procedure, all mice were injected with buprenorphine ER-LAB (1 mg/kg, s.c.; ZooPharm, Laramie, WY, USA). Cells were collected as described above, resuspended in PBS from 2500 to 25,000 cells/µL, depending on the total number of cells injected, and kept on ice throughout the surgeries. Isoflurane anesthesia (induction: 2.5%, room air: 21% O_2_) was delivered with a SomnoSuite^®^ low-flow anesthesia system connected to an induction chamber (Kent Scientific, Torrington, CT, USA). Once anesthetized, mice were transferred to a platform with an infrared warming pad controlled by a RightTemp^®^ temperature monitoring and homeothermic control module (Kent Scientific, Torrington, CT, USA) and positioned into a stereotaxic head frame and anesthesia mask (David Kopf Instruments, Tujunga, CA, USA). We lubricated the eyes with OptixCare^®^ eye lube (Covetrus, Portland, ME, USA), and maintenance anesthesia was set to 1–2% isoflurane for the remainder of the procedure. The shaved scalp was disinfected with a 2% chlorhexidine solution (Covetrus, Portland, ME, USA) and sterile saline (Covetrus, Portland, ME, USA) that were applied alternately using sterile cotton-tipped wood applicators (3 times, 1 min each). Following disinfection of the surgical area, a 1 cm midline incision was made using a 22-blade sterile disposable scalpel (Sklar, West Chester, PA, USA). To remove the remaining periosteum and to visualize bregma, the skull was swabbed with 3% H_2_O_2_ (Ward’s Science, Rochester, NY, USA). A 0.9 mm burr hole was created using an MH-170 rotary handpiece (Foredom Electric Company, Bethel, CT, USA) 2 mm mediolateral and −2 mm anteroposterior from bregma. Once the burr hole was created, cells were gently resuspended and pulled into a 5 µL Hamilton syringe with a 22sG needle (Hamilton Company, Reno, NV, USA). The exterior of the needle was cleaned with an alcohol prep pad.

For the GL261 Red-FLuc model, the needle was slowly inserted over 10 s into the burr hole to a depth of 4 mm, then removed 1 mm to create a pocket for the cells. The GL261 Red-FLuc cell dilution was injected over 2 min (2 µL; 1 µL/min) using an UltraMicroPump 3 with SMARTouch™ Controller (David Kopf Instruments, Tujunga, CA, USA). The needle remained for 1 min, and then was slowly removed over 10 s. Any leakage/blood at the injection site was removed with a cotton-tipped applicator, followed by gentle scrubbing with an EtOH-soaked cotton-tipped applicator to remove any cells that might have made it onto the skull.

The TRP-mCF injection protocol was adapted from El Meskini et al. [25]. The needle was incrementally inserted into the burr hole at a rate of 1 mm/min to a depth of 4 mm, and then retracted 1 mm to create a pocket for the cells. The TRP-mCF cell dilution was injected over 6 min (2 µL; 0.33 µL/min) using an UltraMicroPump 3 with SMARTouch™ Controller (David Kopf Instruments, Tujunga, CA, USA). The needle remained for 1 min, and then was removed incrementally at a rate of 1 mm/min. Any leakage/blood at the injection site was removed with a cotton-tipped applicator, followed by gentle scrubbing with an EtOH-soaked cotton-tipped applicator to remove any cells that might have made it onto the skull. A piece of bone wax (Covetrus, Portland, ME, USA) was shaped into a cone (~1 mm) and placed into the burr hole to prevent any extracranial growth.

For both models, the burr hole was sealed by heating standard pattern forceps (Fine Science Tools, Foster City, CA, USA) with a Germinator 500 Glass Bead Sterilizer (CellPoint Scientific, Gaithersburg, MD, USA) to melt the bone wax (Covetrus, Portland, ME, USA), which was applied over the injection site. The skin was closed with wound clips (Fine Science Tools, Foster City, CA, USA), and the mouse was transferred to a clean cage on a heating pad (Stryker, Kalamazoo, MI, USA). Mice were monitored for at least 3 h post-op until they returned to normal behavior (e.g., movement, eating, drinking, and cleaning). On the days following the injection, mice were observed at least once daily until they reached a humane endpoint (25% bodyweight loss or other adverse clinical signs, such as altered behavior, imbalance, head tilt, or altered respiration. as approved by the IACUC protocol [26,27]).

### 2.4. In Vitro Bioluminescence Imaging

Luciferase expression of GL261 Red-FLuc and TRP-mCF cells was verified using a modified protocol from that previously described [17]. Briefly, 5000, 10,000, 15,000, and 20,000 cells/well were seeded in black clear-bottom 96-well plates (Corning, Corning, NY, USA) and incubated overnight (37 °C, 5% CO_2_). After 24 h, media were aspirated and 100 µL of phenol red-free DMEM (Thermo Fisher Scientific, Waltham, PA, USA) was added to each well. XenoLight^®^ RediJect™ D-luciferin (PerkinElmer, Waltham, MA, USA) was diluted 1:100 in phenol red-free DMEM, and 100 µL of the luciferin dilution (0.15 µg/well) was added to each well. The plate was promptly transferred to an Ami HT optical imaging system (Spectral Instruments Imaging, Tucson, AZ, USA). Bioluminescence was determined by 2D imaging (FOV: 15 cm, exposure time: 1 s, f-stop: 1.2, binning: 4) at the following time points: 1–5, 10, 15, 20, 25, and 30 min. Bioluminescence was quantified using Aura 4.0.7 imaging software (Spectral Instruments Imaging, Tucson, AZ, USA).

### 2.5. In Vivo Bioluminescence Imaging

Bioluminescence imaging of mice harboring luciferase-expressing tumors was conducted weekly to verify tumor take and monitor tumor growth. Mice received 150 mg/kg (5 µL/g; i.p.) of XenoLight^®^ RediJect™ D-luciferin and were anesthetized with 2% isoflurane. At eight min post-luciferin injection, immunocompetent (B6(Cg)-*Tyr^c−2J^*/J) and immunocompromised (J:NU) mice were relocated to the heated imaging stage of either an IVIS^®^ Spectrum in vivo imaging system or a Lago in vivo optical imaging system (Spectral Instruments Imaging, Tucson, AZ, USA), respectively. At ten min post-luciferin injection, tumor bioluminescence was determined by 2D imaging (IVIS-FOV: 21.6 cm, f-stop: 8, binning: 4 or Lago–FOV: 25 cm, f-stop: 2, binning: 2), and images were analyzed using Aura 4.0.7 imaging software (Spectral Instruments Imaging, Tucson, AZ, USA). Tumor doubling times were calculated using a log-linked model, “Biofluorescence ~ ln(Cells) + Week”, where ln(Cells) was the natural logarithm of the cell dose.

### 2.6. Magnetic Resonance Imaging

MRI was conducted at the University of Kentucky Magnetic Resonance Imaging and Spectroscopy Center. Before handling mice, tail vein catheters were prepared by fitting a 29G needle into one side of a Tygon catheter tubing (50″ long; 0.03″ od × 0.01″ id; Braintree Scientific Inc., Braintree, MA, USA) and inserting a 1 mL TB syringe with a 30G needle onto the other end. Sterile saline was loaded into the syringe and perfused through the tubing until saline was expelled from the 29G needle. Mice were anesthetized with 1.5–2% isoflurane, and a tail vein catheter was placed to administer the contrast agent. Once the tail vein catheter was secured, mice were transferred to the platform of a 7 T Bruker BioSpec, small animal MRI scanner (Bruker BioSpin, Billerica, MA, USA). We lubricated the eyes with OptixCare^®^ eye lube (Covetrus, Portland, ME, USA), and a respiration pad transducer and rodent rectal temperature probe were positioned to monitor and document each animal’s vital signs. Pre-contrast T1-weighted (repetition time (TR) = 4000 ms, time to echo (TE) = 2.2 ms, and field of view (FOV) = 20 × 20 × 8 mm) and T2-weighted scans (TR = 4000 ms, TE = 33 ms, and FOV = 20 × 20 × 8) were acquired. Following the acquisition of pre-contrast scans, gadolinium (1 mmol/mL; Gadavist^®^ (gadobutrol), Bayer AG, Whippany, NJ, USA) was diluted 1:10 in sterile saline and administered at a dose of 0.6 mmol/kg through the tail vein catheter. The catheter was flushed with 70 µL of sterile saline to ensure the administration of the full dose of gadolinium (dead volume of 50″ of tubing with a 0.01″ inner diameter ≈ 64 µL). Post-contrast T1-weighted images (same settings as above) were acquired 10 min after the gadolinium injection. Acquired images were analyzed using syngo.via VB60A_HF07 software (Siemens Medical Solutions USA, Inc., Malvern, PA, USA). A conventional T2-RARE sequence was used to identify anatomical features, in conjunction with a standard T1 sequence with administration of a contrast agent to assess contrast enhancement and calculate the tumor volume. The enhanced tumor area was determined using the “Freehand ROI” function, and the total tumor area was calculated using the following equation:

Tumor volume=∑Tumor area∗distance between slices,

where the distance between slices = 400 µm.

### 2.7. Histopathology

Histopathology samples were processed as previously described [17]. At week 3 post-implantation, mice were anesthetized with sodium pentobarbital (150 mg/kg, i.p.) and transcardially perfused with PBS (100 mL, 10 mL/min), followed by perfusion with 10% formalin (50 mL, 10 mL/min; MilliporeSigma, Saint Louis, MO, USA). Mice were decapitated and their brains were removed and placed in 5 mL 10% formalin and stored at RT overnight. The following day, brains were transferred to 70% ethanol and stored at 4 °C until further processing. Samples were dehydrated in increasing concentrations of ethanol (70–100%), defatted with xylene, and embedded in paraffin. Brains were sectioned into two consecutive 4 µm coronal sections at 0.2 mm intervals: one for immunohistochemistry with an anti-luciferase antibody and the other for hematoxylin and eosin (H&E) staining. Decreasing concentrations of ethanol (100–70%) and xylene were used to deparaffinize and rehydrate the tissue, respectively. Anti-luciferase immunohistochemistry was conducted using the Discovery Ultra Research staining system (Ventana Co., Tucson, AZ, USA). Immunohistochemical antigen retrieval was performed using an EDTA-based heat-induced antigen retrieval (CC1) method at 91 °C for 64 min. Slices were stained with an anti-luciferase antibody (ab181640, Abcam, Cambridge, MA, USA) at a 1:250 dilution (4 µg/mL) for 4 h at RT. The antigen–antibody complex was detected using the OmniMap anti-goat multimer RUO detection system and DAB detection kit (Ventana Co., Tucson, AZ, USA). All slices were counterstained with hematoxylin. Brain slices were imaged with an Aperio ScanScope XT (Leica Biosystems, Deer Park, IL, USA) at 20× magnification. The tumor area in each slice was determined manually by outlining the tumor with the Annotations feature of the Aperio ImageScope v12 software (Leica Biosystems Pathology Imaging, Deer Park, IL, USA). The tumor area was also determined using the Deep Learning Classifier Add-On of the HALO^®^ AI analysis platform v3.6 (Indica Labs Inc., Albuquerque, NM, USA). Tumor area was calculated using the following equation:

Tumor volume=∑Tumor area∗(slice thickness+distance between slices),

where the slice thickness + distance between slices = 204 µm.

While the mitotic count and necrosis were assessed using representative H&E-stained slices, perivascular tumor invasion was quantified using representative IHC-stained (anti-luciferase) slices. The mitotic count was assessed using the slice featuring the tumor with the greatest surface area per animal. Using ImageScope, the minimum and maximum x and y coordinates (in pixels) were determined for each tumor slice. Using a random number generator, random x and y coordinates were selected. A 0.16 mm^2^ square ROI, which corresponds to the area of a high-power field [28], was positioned over each central x, y coordinate. ROIs containing the tumor edge were excluded, and the next randomized ROI was analyzed. A total of 5 ROIs was randomly generated for each tumor. Cells with mitotic figures were counted using the “Counter Tool” and summed for all 5 ROIs. Two ordinal scales were defined for perivascular invasion and necrosis (Table 1). Perivascular invasion and necrosis were assessed using a representative IHC- and H&E-stained slide per animal, respectively.

### 2.8. Data Analysis & Statistics

Unless specified otherwise, all data were analyzed by generalized linear mixed-level models (glmer) to account for correlated measurements for individual mice [29].

C57BL/6 mice were modeled with Biofluorescence ~ Cells + Response + Cells × Response, where “Response” was the acceptance or rejection of the tumor. J:NU mice were modeled with Biofluorescence ~ Cells + Week + Cells × Week. The tumor volume vs. analytical method was modeled with Volume ~ Timepoint + Method + Timepoint × Method. Quantitative data were scaled by standard deviation, and predictors were also centered by mean values. Coefficients are, therefore, standardized values. Error families (i.e., Gaussian, gamma, or inverse Gaussian) and link functions (identity or log) were compared by second-order Akaike Information Criterion (AIC) [30]. Specific links are indicated in the results. Survival was modeled with Cox proportional hazards modeling [31] and parametric survival regression [32] using the Akaike Information Criterion with a correction for small sample sizes (AICc) to compare the effects of cell counts as quantities and as levels of a factor under assumptions of proportionality or a specific parametric distribution.

Counts of mitotic activity per ROI were analyzed by generalized linear models using the Conway–Maxwell Poisson distribution [33] to account for possible over or underdispersion. Since areas counted were the same for all animals (0.8 mm^2^), no adjustment was necessary. Both perivascular invasion and necrosis were analyzed by ordered logistic regression [34], and predictions based on these two models were made via estimated marginal means [35]. Coefficients of variation (*R*^2^) were Nakagawa’s [36] for mitotic and Nagelkerke’s [37] for invasiveness and necrosis. R packages used were glmmTMB [38], MASS [39], emmeans [40], and performance [41].

For tumor volume assessments, models were built to determine how methods of volume assessment differed in overall estimates and how well the AI histology and MRI methods agreed with manual assessment of histology. Specifically, for level estimates, we used the mixed-level generalized linear model “Cell type + Method + Cell Type × Method”, with the individual animal as a random intercept. To determine agreement between AI histology or MIR with manual histology, we built parallel linear models of each alternate method vs. manual histology for each cell type. We then used *R*^2^ to quantify the percent linear agreement and compared the square roots of *R*^2^ by the Fisher *z* transformation. Modeling was followed by analysis of deviance (ANOVA) and pairwise comparisons of selected estimated marginal means for level estimates [35]. Coefficients of determination (*R*^2^) were Nagelkerke’s for levels [37] and traditional *R*^2^ for agreement. Partial *R*^2^ for each effect in the level estimate model were estimated by calculating *R*^2^ for nested sub-models lacking an effect and subtracting this from the overall model *R*^2^. Omnibus (overall) ANOVAs for level estimates were χ^2^ tests of models vs. intercept only or intercept and animal random effects only. The R environment [42] was used to perform analyses with the lme4 [43] and emmeans [40] packages.

## 3. Results

### 3.1. In Vitro Verification of Luciferase Activity

**Figure 1:**
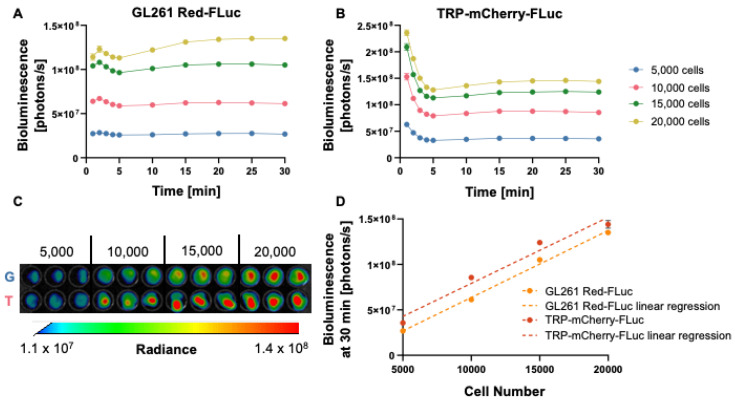
In vitro verification of luciferase activity. (**A**) Quantified in vitro bioluminescence of 5000; 10,000; 15,000; and 20,000 GL261 Red-FLuc cells from 1 to 30 min following the addition of D-luciferin (0.5 µg/well) (*n* = 3, 3 technical replicates; data points represent means ± SEMs). (**B**) Quantified in vitro bioluminescence of 5000; 10,000; 15,000; and 20,000 TRP-mCF cells from 1 to 30 min following the addition of D-luciferin (0.5 µg/well) (*n* = 3, 3 technical replicates; data points represent means ± SEMs). (**C**) Representative image of in vitro bioluminescence of GL261 Red-FLuc (G) and TRP-mCF (T) cells 30 min after the addition of D-luciferin. (**D**) Linear correlation between the seeded cell number and bioluminescence for GL261 Red-FLuc and TRP-mCF cells (GL261 Red-FLuc: Y = 7366 * X – 10,058,920, *R*^2^ = 0.985, *p* < 0.0001; TRP-mCF: Y = 7268 * X + 6,461,663, *R*^2^ = 0.932, *p* < 0.0001). Statistics: Simple linear regression.

We confirmed the luciferase activity of the luciferase-expressing GL261 Red-FLuc and TRP-mCherry-FLuc cell lines through in vitro bioluminescence imaging (Figure 1A,B). Bioluminescence of GL261 Red-FLuc cells peaked 2 min after adding luciferin, slightly decreased through 5 min, then increased, and stabilized from 5 to 30 min, depending on the cell number. Bioluminescence of TRP-mCF cells was highest 1 min after adding luciferin, decreased through 5 min, then increased slightly, and stabilized from 5 to 30 min. After 30 min, stable bioluminescence was observed, showing a linear correlation between the cell number and bioluminescence for both cell lines (GL261 Red-FLuc: *R*^2^ = 0.985, *p* < 0.0001; TRP-mCF: *R*^2^ = 0.932, *p* < 0.0001) (Figure 1C,D). This suggests that as cell numbers increase, bioluminescence production rises consistently and without a plateau, which is important for tracking tumor growth in vivo. These data verify that both GL261 Red-FLuc and TRP-mCF express active luciferase.

### 3.2. GL261 Red-FLuc Tumor Take and Survival in Immunocompetent (Albino B6) Mice

**Figure 2:**
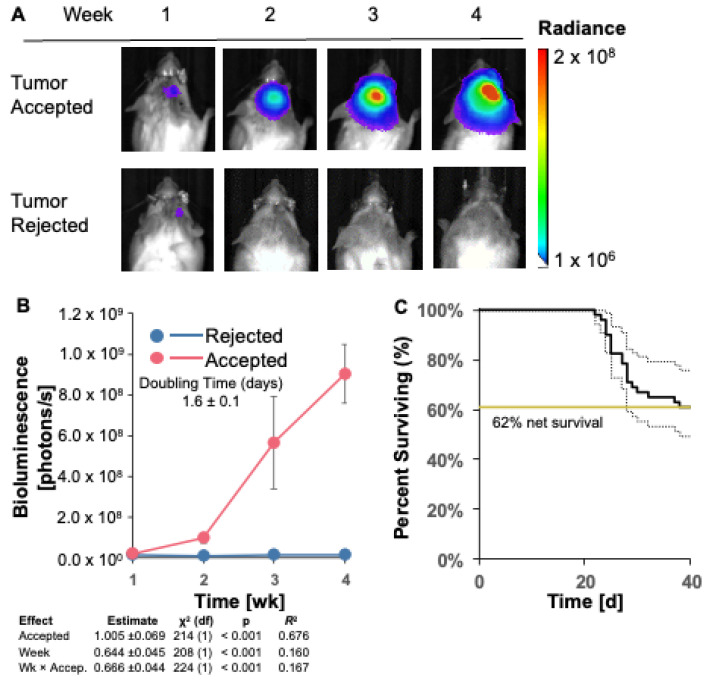
GL261 Red-FLuc tumor take and survival in immunocompetent (albino B6) mice. (**A**) Representative in vivo bioluminescence images of albino B6 mice intracranially injected with 50,000 GL261 Red-FLuc cells. Total emission range: 1 × 10^6^ to 2 × 10^8^ photons/s. (**B**) Quantified bioluminescence of accepting and rejecting mice over 4 weeks post-implantation (above) with descriptive statistics of modeled data (below). Coefficient estimates are on log link. *R*^2^ is for fixed effects. Data points represent means ± SEMs. (**C**) Survival curves of mice with accepted or rejected intracranial GL261 Red-FLuc tumors. Gray lines represent the standard error of the function (*n* = 51).

To establish luciferase-expressing GL261 Red-FLuc tumors in immunocompetent hosts, we intracranially implanted 5 × 10^4^ GL261 Red-FLuc cells into 8-week-old female albino C57BL/6 mice. We verified the successful implantation of cells into mouse brains one week after injection and tracked tumor growth weekly through bioluminescence imaging using the IVIS^®^ Spectrum in vivo imaging system. Figure 2A,B shows representative images and quantified bioluminescence for albino C57BL/6 mice at 1–4 weeks post-implantation. Mice with successful tumor take throughout the end of the study (40 days post-implantation) are labeled as “accepted”, and mice with no evidence of a tumor by the end of the study are labeled as “rejected”. Only 22 of 51 (43.1%) mice showed evidence of a tumor two weeks after implantation, which decreased to 20 of 51 (39.2%) by week 3. The doubling time of accepted tumors was 1.6 ± 0.1 days (Figure 2B and Table 2). By the end of the study, the mortality was 38% ± 15/12% (Figure 2C; SE estimated by the Cox model of survival vs. intercept). In contrast, tumors were rejected in 31 of 51 (61%) of mice, which is consistent with previous reports using GL261 Red-FLuc cells in immunocompetent mice [20] (Figure 2A–C). The median survival of mice that accepted tumors and reached the endpoint was 27 days (Figure 2C and Table 2). In summary, most GL261 Red-FLuc tumors are rejected in immunocompromised mice, making this a suboptimal model for GBM research.

### 3.3. GL261 Red-FLuc Tumor Take and Survival in Immunocompromised (J:NU) Mice

**Figure 3:**
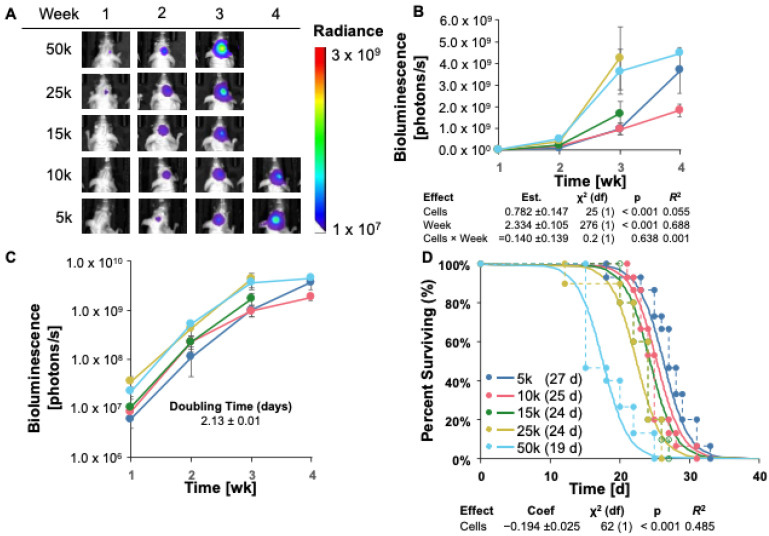
GL261 Red-FLuc tumor take and survival in immunocompromised (J:NU) mice. (**A**) Representative images of tumor bioluminescence in J:NU mice intracranially injected with GL261 Red-FLuc cell doses ranging from 5000 to 50,000 cells. Total emission range: 1 × 10^7^ to 3 × 10^9^ photons/s. (**B**) Quantified bioluminescence of mice by dose and week post-implantation (above) and descriptive statistics of modeled data (below). Coefficients estimated on log link. (**C**) Log-response of quantified bioluminescence by dose and week post-implantation. (**D**) Survival modeling of mice by dose and days post-implantation (above) and descriptive statistics of modeled data (below). Solid lines show the predicted effects of the cell dose (logistic link). Points joined by dashed lines are Kaplan–Meier curves segregated by dose. Numbers in parentheses in the legend represent median survival in days. Statistics (χ^2^, *p*, *R*^2^) are from the parametric survival model. Data points represent means ± SEMs. For group numbers, see Table 3.

Given the high rate of tumor regression in albino B6 mice, we sought to increase tumor engraftment by implanting GL261 Red-FLuc cells into immunocompromised, female athymic nude (J:NU) mice. The injection procedure for luciferase-expressing cells in J:NU mice was identical to C57BL/6 mice. To test survival, the number of intracranially implanted cells ranged from 5000 to 50,000. Representative bioluminescence images and average bioluminescence quantifications are shown in Figure 3A and Figure 3B, respectively. No representative images are shown for mice implanted with 15,000, 25,000, or 50,000 cells at week 4 since most mice had already reached the endpoint.

Tumor growth, measured by bioluminescence, appeared to have been in the log phase in weeks 1–3 (Figure 3B,C). Therefore, explicit modeling was restricted to these time points. We used a log-linked model, “Biofluorescence ~ ln(Cells) + Week”, where ln(Cells) was the natural logarithm of the number of cells implanted. This model was significant for both variables (cells and week; Figure 3B); however, there was no significant interaction between the cell number and time, suggesting that the tumor doubling times for each injected cell number cannot be presumed to differ significantly. With this model, we estimated the average tumor doubling time as 2.13 ± 0.1 days (Figure 3C and Table 3).

When we compared different survival models, AICc favored a parametric survival vs. cell dose model with a logistic distribution (Figure 3D). Specifically, as we increased the number of injected GL261 cells, survival decreased, as expected. Unlike in the albino C57BL/6 mice, all starting cell numbers yielded 100% tumor take in J:NU mice. A direct comparison of survival rates between immunocompetent (albino B6) and immunocompromised (J:NU) implanted with 50,000 GL261 Red-FLuc cells is depicted in Figure S2. Due to this 100% tumor take and the longest survival of the starting cell numbers, we injected J:NU mice with 5 × 10^3^ GL261 Red-FLuc cells in all remaining studies.

### 3.4. TRP-mCherry-FLuc Tumor Take and Survival in Immunocompetent (Albino B6) Mice

Since the engraftment of GL261 Red-FLuc tumors in immunocompetent mice was low, we aimed to assess the tumor take of another luciferase-expressing cell line, TRP-mCF. Notably, TRP-mCF cells behave more aggressively than GL261 Red-FLuc in vitro, demonstrating shorter doubling times when seeded at cell densities up to 4000 cells (Figure S1; Table S1). Therefore, we hypothesized that these more proliferative cells could overcome the immunoreactive microenvironment of immunocompetent mice.

**Figure 4:**
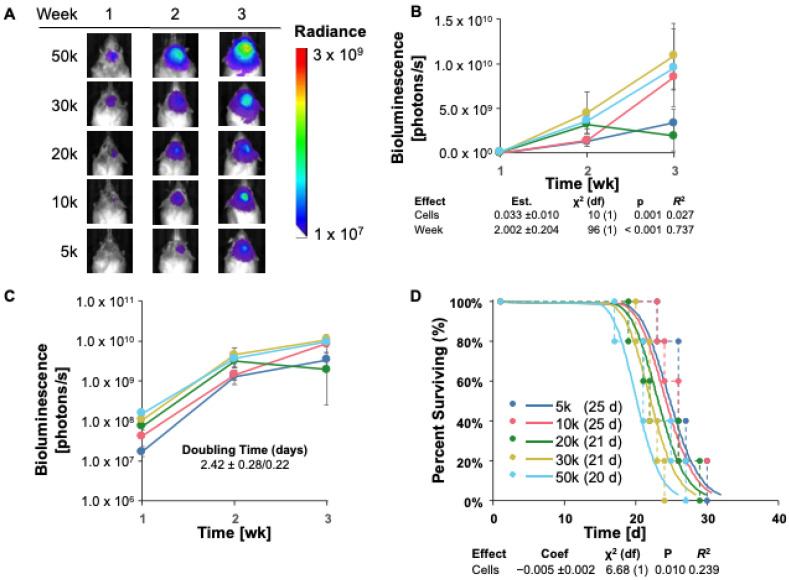
TRP-mCherry-FLuc tumor take and survival in immunocompetent (albino B6) mice. (**A**) Representative images of tumor bioluminescence in albino B6 mice intracranially injected with TRP-mCF cell doses ranging from 5000 to 50,000 cells. Total emission range: 1 × 10^7^ to 3 × 10^9^ photons/s. (**B**) Quantified bioluminescence of mice by dose and week post-implantation (above) and descriptive statistics of modeled data (below). Coefficients estimated on log link. (**C**) Log-response of quantified bioluminescence by dose and week post-implantation. (**D**) Survival modeling of mice by dose and days post-implantation (above) and descriptive statistics of modeled data (below). Solid lines show the predicted effects of the cell dose (logistic link). Points joined by dashed lines are Kaplan–Meier curves segregated by dose. Numbers in parentheses in the legend represent median survival in days. Statistics (χ^2^, *p*, *R*^2^) are from the parametric survival model. Data points represent means ± SEMs (*n* = 5/group).

Analogous to GL261 Red-FLuc cells, we intracranially implanted 5 × 10^3^ to 5 × 10^4^ TRP-mCF cells into 8-week-old female albino C57BL/6 mice. We verified successful cell implantation into mouse brains one week after injection and tracked tumor growth weekly through bioluminescence imaging using the IVIS^®^ Spectrum in vivo imaging system. Representative weekly bioluminescence images and average quantified bioluminescence are shown in Figure 4A and Figure 4B, respectively. Most mice reached the prespecified endpoint during week 3, with tumor bioluminescence no longer detectable in one of the five mice injected with 10,000 cells by the second week post-implantation. As measured by bioluminescence, tumor growth appeared to have been in the log phase from weeks 1 to 3 (Figure 4A–C). A log-linked model, “Biofluorescence ~ Cells + Week”, was significant for both variables, suggesting that cell number did affect the weekly tumor size (Figure 4B). A potential model without the interaction of the cell count and time was more likely by AICc comparison than one with this interaction, suggesting that the impact of the cell number on bioluminescence (tumor size) was consistent across time points and vice versa. This model was also used to estimate the doubling time as 2.42 ± 0.28/0.22 days (Figure 4C and Table 4).

When different survival models were compared, a parametric model of survival vs. cell dose with a lognormal distribution (Figure 4D) was favored by AICc. Specifically, as the cell dose increased, survival decreased in a rational fashion. Overall, these data highlight that TRP-mCherry-FLuc tumors can overcome the immunosuppressive microenvironment of albino B6 mice, yielding 100% tumor take with most cell numbers injected.

### 3.5. MRI Features and Tumor Volume Determination

**Figure 5:**
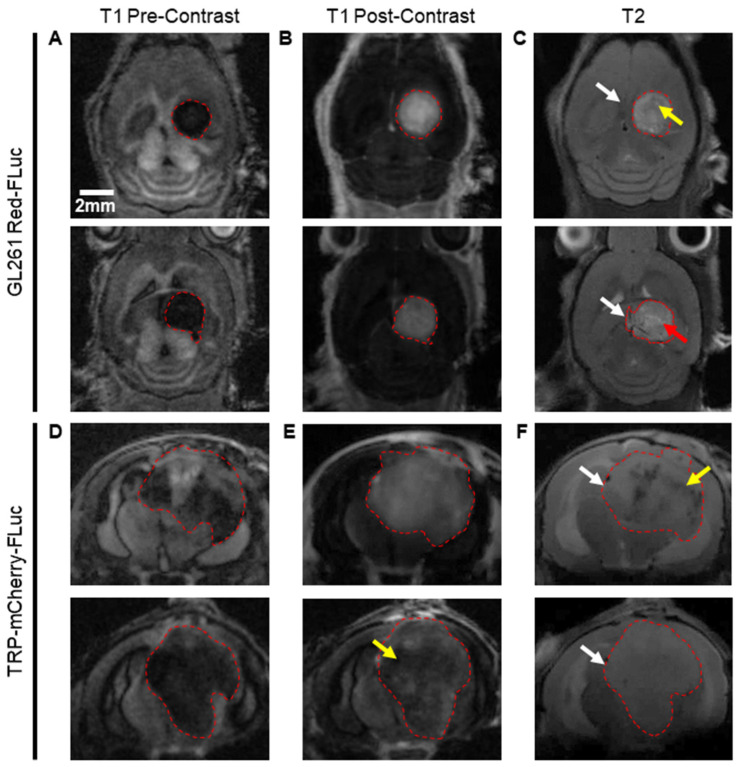
MRI features and tumor volume determination. Top: Representative (**A**) pre-contrast T1-weighted, (**B**) post-contrast T1-weighted, and (**C**) T2-weighted images of J:NU mice injected with 5000 GL261 Red-FLuc cells. Bottom: Representative (**D**) pre-contrast T1-weighted, (**E**) post-contrast T1-weighted, and (**F**) T2-weighted images of albino B6 mice injected with 5000 TRP-mCF cells. Images were taken at three weeks post-intracranial injection. Red arrow: light region (fluid); yellow arrow: dark region (hematoma) or low Gd contrast enhancement; white arrow: mass effect. Size bar = 2 mm.

We imaged J:NU and albino B6 mice injected with 5 × 10^3^ GL261 Red-FLuc and TRP-mCF cells using MRI at three weeks post-implantation. Representative MR images for two animals of each model are shown in Figure 5.

GL261 Red-FLuc tumors were evident on pre-contrast T1-weighted, post-contrast T1-weighted, and T2-weighted images, and the average tumor volume was 28.9 ± 18.8 mm^3^. On pre-contrast T1-weighted images, tumors appeared as well-demarcated hypodense masses in the right hemisphere (Figure 5A). Following the i.v. injection of a gadolinium (Gd)-based contrast agent, tumors displayed heterogenous contrast enhancement, signifying blood–brain barrier disruption (Figure 5B). T2-weighted images showed well-demarcated masses with light (red arrow) and dark (yellow arrow) regions, suggesting the presence of fluid and hematoma, respectively (Figure 5C). An appreciable mass effect (white arrows) was seen in all mice, with involvement of the contralateral hemisphere in larger tumors. Similarly, TRP-mCF tumors were apparent on pre-contrast T1-weighted, post-contrast T1-weighted, and T2-weighted images, with an average tumor volume of 109.5 ± 38.9 mm^3^. Tumors were less demarcated than GL261 Red-FLuc tumors on pre-contrast T1-weighted images, with cloudy tumor borders, suggesting tumor infiltration and/or peritumoral changes (Figure 5D). Moderate contrast enhancement was observed on post-contrast T1-weighted images, with some tumors exhibiting diminished enhancement in their central regions (yellow arrow), suggesting necrosis, hemorrhage, an increased cell density, or increased interstitial pressure in these areas (Figure 5E). On T2-weighted images, tumor borders were less discernable, and there was a larger mass effect (white arrows) compared to GL261 Red-FLuc tumors (Figure 5F). Some tumors contained dark regions (yellow arrow), suggesting the presence of hematoma.

These data show that GL261 Red-FLuc tumors manifest as circular, well-demarcated masses, while TRP-mCF tumors exhibit increased signs of invasion, along with necrotic and/or densely compact cores.

### 3.6. Immunohistochemical Features of GL261 Red-FLuc and TRP-mCherry-FLuc Tumors

**Figure 6:**
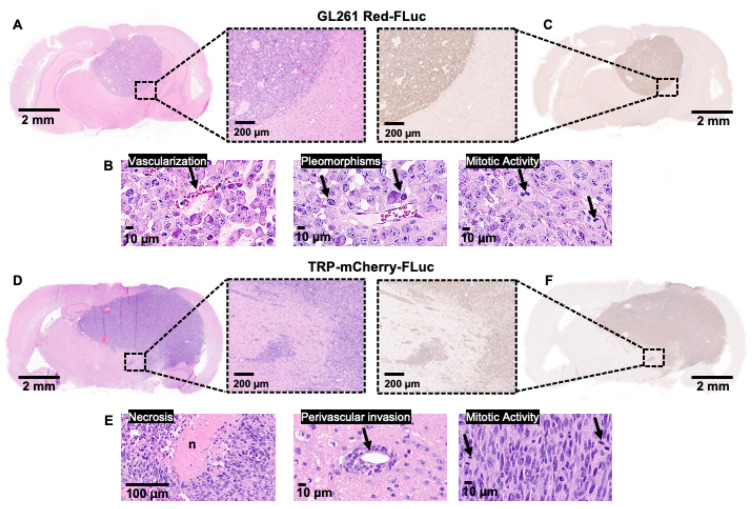
Immunohistochemical features of GL261 Red-FLuc and TRP-mCherry-FLuc tumors. (**A**) Representative H&E-stained section of a GL261 Red-FLuc tumor from a J:NU mouse. Size bars = 2 mm (full slice) and 200 µm (11× zoom). (**B**) Close-up images of GL261 Red-FLuc H&E showing vascularization, pleomorphisms, and enhanced mitotic activity. Size bar = 10 µm. (**C**) Representative anti-firefly luciferase IHC staining of a GL261 Red-FLuc tumor from a J:NU mouse. Size bars = 2 mm (full slice) and 200 µm (11× zoom). (**D**) Representative H&E-stained section of a TRP-mCF tumor from an albino B6 mouse. Size bars = 2 mm (full slice) and 200 µm (11× zoom). (**E**) Close-up images of TRP-mCF H&E showing necrosis, perivascular tumor invasion, and enhanced mitotic activity. Size bars = 100 µm (left panel) and 10 µm (middle and right panels). (**F**) Representative anti-firefly luciferase IHC staining of a TRP-mCF tumor from an albino B6 mouse. Size bars = 2 mm (full slice) and 200 µm (11× zoom).

**Figure 7:**
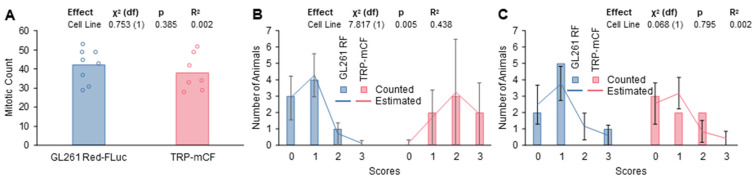
Mitotic count, perivascular invasiveness, and necrosis of subject animals. Representative slides of animal brains were rated for (**A**) the mitotic count, (**B**) perivascular invasiveness score, and (**C**) necrosis. For the mitotic count, the number of cells undergoing mitosis were quantified and combined for 5 randomized ROIs per animal (0.16 mm^2^/ROI; 0.8 mm^2^ total area). For invasiveness and necrosis scores, bars represent measurements and lines represent least square mean estimates from the regression with estimated standard errors. *n* = 8 animals (GL261 Red-FLuc) and 7 animals (TRP-mCF).

To verify the characteristics observed on MRI with increased resolution, we transcardially perfused and fixed the brains of nude and albino B6 mice injected with 5 × 10^3^ GL261 Red-FLuc or TRP-mCherry-FLuc cells, respectively, at three weeks post-implantation. For GL261 Red-FLuc tumors, histological morphology assessed using H&E staining demonstrated densely packed cells with high mitotic activity, nuclear pleomorphisms, and vascularization (Figure 6A,B). Similar to what we observed by MRI, tumors were well-circumscribed with minimal invasive cells, similar to the characterization of parental GL261 tumors in immunocompetent C57BL/6 mice [23,44,45]. IHC revealed strongly stained luciferase-positive cells with a well-demarcated border (Figure 6C). At the same time point post-implantation, TRP-mCF tumors occupied most of the right hemisphere, causing a significant mass effect, with many tumors invading the contralateral hemisphere (Figure 6D). H&E staining displayed areas of necrosis, perivascular tumor invasion, and increased mitotic activity (Figure 6E). Unlike GL261 Red-FLuc tumors, invasive cell nests were seen around the main tumor border, which was verified using anti-luciferase IHC (Figure 6F). Comparing the overall profiles of perivascular invasion scores (Figure 7B) by ordered logistic regression showed a significant difference (χ^2^ = 7.817, *p* = 0.005). Specifically, GL261 Red-FLuc tumors showed less invasiveness than did TRP-CF tumors. Profiles of score frequencies took a “mirror” approach vs. each other. On the other hand, no significant differences were found in mitotic counts (Figure 7A) or necrosis scores (Figure 7C).

### 3.7. High-Throughput Determination of GL261 Red-FLuc and TRP-mCF Tumor Volumes and Comparison to MRI

**Figure 8:**
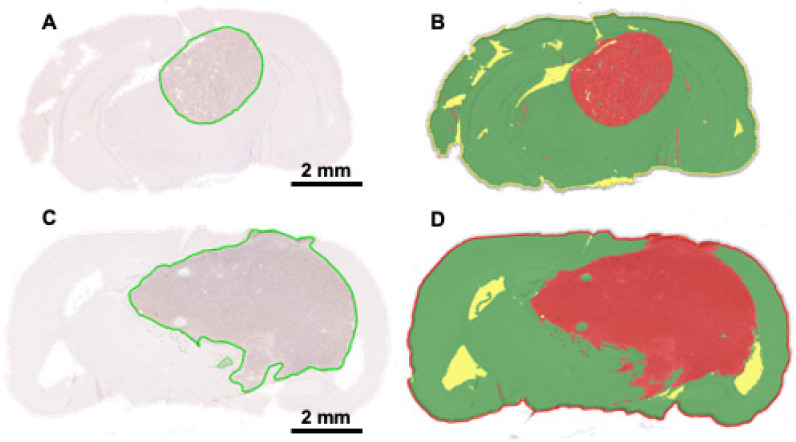
High-throughput determination of GL261 Red-FLuc and TRP-mCF tumor volumes. (**A**) Representative anti-firefly luciferase immunohistochemistry of GL261 Red-FLuc tumors from J:NU mice injected with 5000 cells at three weeks post-implantation. (**B**) Analysis of the tumor area determined by the HALO AI™ Deep Learning Classifier Add-On. Red = tumor; green = non-tumor; yellow = glass/background. (**C**) Representative anti-firefly luciferase immunohistochemistry of TRP-mCF tumors from albino B6 mice injected with 5000 cells at three weeks post-implantation. (**D**) Analysis of the tumor area determined by the HALO AI™ Deep Learning Classifier Add-On. Red = tumor; green = non-tumor; yellow = glass/background.

**Figure 9:**
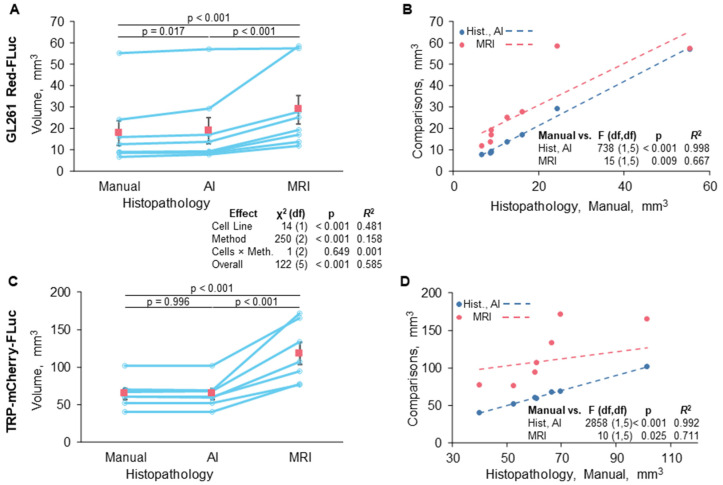
Comparison of tumor volume estimation methods. (**A**,**B**) GL261 Red-FLuc and (**C**,**D**) TRP-mCF tumor volumes were measured by manual and AI quantification of histopathology samples and by MRI. We compared (**A**,**C**) differences in overall estimated levels and (**B**,**D**) agreement between methods. Rose-colored squares = means ± SEMs.

We also calculated tumor volumes from histopathology to verify volumes determined by MRI. The tumor area of each slice was calculated manually using Aperio ImageScope v12 software and the HALO^®^ v3.6 AI Deep Learning Classifier Add-On. Examples of manually analyzed anti-luciferase IHC-stained slices are shown in Figure 8A,C, with tumors outlined in green. Examples of slices analyzed using AI are shown in Figure 8B,D, with red, green, and yellow corresponding to the tumor, non-tumor, and glass/background, respectively. The total tumor volume was calculated by multiplying the sum of the tumor area by the distance between the slices for each mouse. Average GL261 Red-FLuc and TRP-mCF tumor volumes, as determined from histopathology slices, are listed in Table 5. Paired tumor volumes, determined manually and with AI from histopathology samples, are compared to MRI in Figure 9.

Mean tumor volumes were similar from both manual and AI interpretations of histopathology (Figure 9A,C). There were some significant (*p* = 0.017) but small elevations in the estimates for GL261 Red-FLuc tumors. These observed disparities may be attributed to the detection of minor artifacts and darker staining regions (e.g., the hippocampus). On the other hand, tumor volume estimates were noticeably higher overall for MRI (Figure 9A,C), particularly for TRP-mCF. There were two potential visual outliers in the GL261 Red-FLuc readings, but when we examined normalized Pearson residuals, no points deviated sufficiently from the model predictions to consider rejection. The difference between MRI and histopathology was further underlined when comparing the linear agreement between AI and manual assessments of histopathology and between MRI and manual assessments of histopathology (Figure 9B,D), arbitrarily choosing the manual assessment as a “standard”. For both GL261 Red-FLuc and TRP-mCF tumors, the agreement (as *R*^2^) was >99%. On the other hand, the agreement between manual histopathology and MRI was only 71% for GL261 and 67% for TRP. When we performed a *z* test on Fisher-transformed square roots of *R*^2^, both differences were significant (GL261 *z* = 2.959, *p* = 0.002; TRP *z* = 3.846, *p* < 0.001). ANOVAs of the models were significant (*p* ≤ 0.05), but they were trivial since the relationships were mostly monotonic.

## 4. Discussion

Preclinical models that accurately replicate key features of human GBM serve as a valuable platform for investigating tumor biology, testing novel therapies, and predicting clinical responses. These models play a critical role in bridging the gap between preclinical studies and human trials, contributing to the development of more precise and effective interventions compared to the standard of care for GBM. In preclinical studies, luciferase-tagged GBM cells are used to monitor tumor growth and the treatment response in animals, allowing non-invasive in vivo imaging. However, recent reports have highlighted inconsistent tumor engraftment of luciferase-tagged cells in immunocompetent mice, rendering these models unreliable for high-throughput studies. While the immunogenicity of luciferase-expressing cell lines is an established phenomenon, it is largely underreported yet an important consideration for model selection. This is particularly crucial, considering the aim to “reduce” animal numbers in the 3Rs of research, as a significant number of mice could be deemed “wasted” when other research groups optimize luciferase-expressing GBM models in their laboratories. Consequently, further optimization and validation of these models are imperative to ensure their reliability and suitability for research purposes [20,46].

Among the syngeneic models used in GBM research, the GL261 model stands out as the most used due to its high reproducibility and shared pathological and molecular features with human GBM [9,10]. However, this model has limitations, as it lacks other features of high-grade gliomas, including invasive cells, vessel co-option, and pseudopalisading necrosis. The survival of mice injected with varying concentrations of parental GL261 cells is reported by Szatmári et al. [9], which we relied on when optimizing the GL261 model. However, no such reports are published for luciferase-expressing GL261 tumors. We believe our manuscript impacts the broader GBM community by providing a comprehensive analysis of the cell dose–survival response of the GL261 Red-FLuc model, alongside detailed and reproducible methods to achieve 100% tumor engraftment.

Here, we validate earlier findings of GL261 Red-FLuc immunoreactivity in immunocompetent mice, present an alternative approach with highly detailed and reproducible methods to improve tumor take, and characterize the GL261 Red-FLuc tumor growth rate and survival. We found that GL261 Red-FLuc tumors spontaneously regressed in more than 60% of immunocompetent (albino B6) mice injected with 5 × 10^4^ GL261 Red-FLuc cells. These data are consistent with previous work by Sanchez et al. that demonstrates spontaneous regression and long-term survival (>100 days) in 60% of C57BL/6 mice [20]. This phenomenon is most likely due to the immunogenicity of the Red-FLuc tag, which induces a proinflammatory microenvironment with increased macrophage and T-cell infiltration compared to parental, untagged GL261 cells, leading to GBM implant rejection.

Variable tumor growth and spontaneous tumor regression are not suitable characteristics of a reliable preclinical model due to the inability to distinguish between treatment effects and immune-mediated tumor regression. Therefore, we aimed to generate a luciferase-expressing GL261 model with consistent tumor growth. Using immunocompromised mice (J:NU nude), we achieved 100% tumor take rate following the implantation of 5000–50,000 GL261 Red-FLuc cells. Increasing cell numbers led to a cell number-dependent decrease in median survival, ranging from 19 to 27 days. Szatmári et al. [9], who used the parental GL261 model in immunocompetent C57BL/6 mice, revealed similar survival rates, with median survivals of 27 and 25 days for mice implanted with 1 × 10^4^ and 1 × 10^5^ cells, respectively. On the other hand, we show that cells with a more aggressive phenotype, TRP-mCF, can overcome the immunoreactivity of firefly luciferase to achieve high tumor engraftment in immunocompetent mice. The TRP model is currently an underutilized but highly relevant GBM model, given its high degree of proliferation, invasiveness, and vascularity. We provide the most detailed characterization of the luciferase-expressing TRP cell line to date in the hopes that others will take advantage of this model. This homograft model combines the advantages of genetically engineered mouse models, featuring mutations and histopathological features consistent with human tumors, with the added benefits of more consistent tumor take and shorter lag time for tumor growth. These attributes are better suited for high-throughput studies and enhance the model’s utility for preclinical investigations.

Data from our MRI and histopathological analyses of GL261 Red-FLuc tumors in immunocompromised mice are consistent with the features of parental GL261 tumors in immunocompetent mice [8,45,47]. On day 21 post-implantation, GL261 Red-FLuc tumors exhibited well-demarcated borders with a small degree of edema and demonstrated heterogeneous contrast enhancement on post-Gd T1-weighted images. Tumors showed vascular proliferation, high cellularity, nuclear pleomorphism, high mitotic activity, and distinct borders, which is a characteristic feature of carcinogen-induced mouse gliomas [48]. Relative to GL261 Red-FLuc tumors, TRP-mCF tumors were much larger by day 21 and displayed increased evidence of necrosis, edema, and contralateral hemisphere involvement on MRI. On histopathology, TRP-mCF tumors were invasive and exhibited necrosis, vessel co-option, and high mitotic activity. Compared to GL261 Red-FLuc tumors, TRP-mCF tumors had significantly higher perivascular invasiveness scores but similar mitotic activity and necrosis.

Alongside descriptive histopathology, we also introduce an AI-based approach for automating the tumor volume analysis. The automatization of the histopathological analysis has advantages, including increased efficiency, consistency, standardization, reduced subjectivity, and the ability to handle large datasets. We trained HALO’s AI™ Deep Learning Classifier Add-On to classify regions as glass (background), non-tumor, or tumor tissue on slides stained for firefly luciferase. On day 21 post-implantation, the tumor volume was assessed manually, with noticeable differences observed when employing AI. However, these differences were minimal (GL261 Red-FLuc: 17.7 ± 16.3 mm^3^ vs. 19.0 ± 17.0 mm^3^; TRP-mCF: 64.4 ± 19.0 mm^3^ vs. 64.4 ± 19.1 mm^3^ for manual and AI calculations, respectively) and could be attributed to factors such as the small sample size (*n* = 7–8 tumors) or limitations of AI in accurately discerning necrotic regions, which may resemble glassy backgrounds or dark brain regions with a high cellular density resembling the tumor tissue. Furthermore, calculated tumor volumes differed significantly when determined by histopathology and MRI; however, this discrepancy is consistent with previous studies comparing brain tumor volumes using these methods [17,49,50]. These differences could be due to various factors, including resolution differences, tissue processing with either shrinkage artifacts, or delicate tumor fragments prone to detachment, tumor edema, or imaging artifacts. Regardless, these differences suggest that tumor volume quantification via histopathology and MRI are not interchangeable and should be consistent within a study.

We acknowledge the potential concern regarding the use of immunodeficient mice with syngeneic tumor models, especially considering the availability of human tumor models. However, the level of immunodeficiency in J:NU mice is not as severe as in other immunocompromised strains, such as NCG and NOD SCID. J:NU mice lack T cells, but B cells, dendritic cells, macrophages, natural killer cells, and hemolytic complement are present. Furthermore, our characterization of the GL261 Red-FLuc tumors in J:NU mice shows that the tumors maintain histopathological features of parental GL261 tumors in C57BL/6 mice reported in the literature [8,45,47]. There are also benefits and contexts where using syngeneic models in immunocompromised mice can provide valuable insights in preclinical research, allowing a focused examination of tumor biology without immune interference. For example, researchers have implanted GL261 tumors in immunocompromised mice to eliminate adaptive immunity as a variable [51,52]. Ruotsalainen et al. compared the efficacy of VA7 virotherapy against GL261 tumors in both C57BL/6 and athymic C57BL/6 mice, showing similar treatment responses between models [52]. These researchers also demonstrated that interferon-β did not have cross-species reactivity, suggesting that the effectiveness of human tumors implanted within the mouse interferon microenvironment has limited relevance for translational investigations focused on oncolytic virotherapy. Similarly, Kober et al. implanted GL261 cells into athymic Balb/c athymic, athymic C57BL/6, and wild-type C57BL/6 mice, which allowed them to demonstrate the involvement of the adaptive immune system as a microenvironmental modulator [51]. Furthermore, Thotala et al. showed improved survival with valproic acid and radiation in GL261-GFP-FLuc tumors in immunocompromised mice, translating to clinical trials with survival benefits [53,54]. These findings underscore the value of such models in advancing translational research.

Overall, our study demonstrates the challenges and opportunities associated with optimizing and validating luciferase-expressing GBM models, emphasizing the importance of selecting the most appropriate model for specific research questions and experimental objectives.

## 5. Conclusions

By characterizing the GL261 Red-FLuc model in J:NU mice, we have addressed the limitations of inconsistent tumor take observed in C57BL/6 mice, providing a model useful for studies either focusing on direct cytotoxic effects of therapies or without the confounding effects of the immune system. We also demonstrate that other luciferase-expressing models, like TRP-mCF, can yield high tumor engraftment in immunocompetent hosts. In addition, while acknowledging the challenges in AI-based tumor volume analysis, we use these models to demonstrate their potential to complement traditional methods of tumor volume analysis, yielding values similar to those of manual analyses while saving time and effort. In conclusion, we demonstrate how to achieve high GL261 Red-FLuc and TRP-mCF tumor take to track tumor growth and the treatment response reliably in GBM research without experimental variability. Since each model has strengths and weaknesses, selecting the appropriate preclinical model should be based on the project goals.

## Figures and Tables

**Table 1:** Perivascular invasiveness and necrosis scoring criteria.

Score	Invasiveness	Necrosis
0	None	None
1	<5 vessels/slide with associated tumor cells	<5 focal necrotic regions
2	>5 vessels/slide with associated tumor cells + no involvement of vessels > 500 µm from the tumor edge	>5 focal necrotic regions
3	>5 vessels/slide with associated tumor cells + involvement of vessels > 500 µm from the tumor edge	confluent necrosis involving larger, continuous areas of the tumor

**Table 2:** In vivo tumor growth characteristics of immunocompetent mice injected with 50,000 GL261 Red-FLuc cells.

Tumor Take	Doubling Time (d) ^1^	Median Survival (d)	N
Accepted	1.6 ± 0.1	27	20
Rejected	N/A	N/A	31

^1^ Tumor doubling times were calculated using a log-linked model, “Biofluorescence ~ ln(Cells) + Week”, where ln(Cells) was the natural logarithm of cell dose. Values are presented as means ± SDs.

**Table 3:** In vivo tumor growth characteristics of immunocompromised mice injected with decreasing numbers of GL261 Red-FLuc cells.

Cell Number	Doubling Time (d) ^1^	Median Survival (d)	N
50k	2.7 ± 0.5/0.7	19	17
25k	2.4 ± 0.2/0.2	24	10
15k	2.3 ± 0.1/0.1	24	10
10k	2.2 ± 0.1/0.1	25	15
5k	2.2 ± 0.2/0.1	27	15

^1^ Tumor doubling times were calculated using a log-linked model, “Biofluorescence ~ ln(Cells) + Week”, where ln(Cells) was the natural logarithm of cell dose. Values are presented as means ± SDs.

**Table 4:** In vivo tumor growth characteristics of mice injected with decreasing numbers of TRP-mCherry-FLuc cells.

Cell Number	Doubling Time (d) ^1^	Median Survival (d)	N
50 k	2.4 ± 0.4/0.7	20	5
30 k	2.4 ± 0.3/0.3	21	5
20 k	2.4 ± 0.2/0.3	21	5
10 k	2.4 ± 0.3/0.4	25	5
5 k	2.4 ± 0.3/0.4	25	5

^1^ Tumor doubling times were calculated using a log-linked model, “Biofluorescence ~ ln(Cells) + Week”, where ln(Cells) was the natural logarithm of cell dose. Values are presented as means ± SDs.

**Table 5:** GL261 Red-FLuc and TRP-mCF tumor volume as determined from histopathology and MRI.

Model	Tumor Volume (mm^3^) as Determined by			N
	Manual	AI	MRI	
GL261 Red-FLuc	17.7 ± 16.3	19.0 ± 17.0	28.9 ± 18.8	8
TRP-mCF	64.4 ± 19.0	64.4 ± 19.1	109.5 ± 38.9	7

## Data Availability

The original contributions presented in the study are included in the article/Supplementary Materials; further inquiries can be directed to the corresponding author.

## Supplementary Materials

The following supporting information can be downloaded at https://www.mdpi.com/article/10.3390/cancers16111997/s1: Figure S1. Comparison of GL261 Red-FLuc and TRP-mCF in vitro growth characteristics; Figure S2. Survival comparison of immunocompetent (albino B6) and immunocompromised (J:NU) mice injected with 50,000 GL261 Red-FLuc cells. Table S1. In vitro GL261 Red-FLuc and TRP-mCF growth characteristics; and Table S2. Experiment summary.

## References

[1] Ostrom Q.T., Price M., Neff C., Cioffi G., Waite K.A., Kruchko C., Barnholtz-Sloan J.S. (2023). CBTRUS Statistical Report: Primary Brain and Other Central Nervous System Tumors Diagnosed in the United States in 2016-2020. Neuro Oncol..

[2] Robin A.M., Pawloski J.A., Snyder J.M., Walbert T., Rogers L., Mikkelsen T., Noushmehr H., Lee I., Rock J., Kalkanis S.N. (2022). Neurosurgery’s Impact on Neuro-Oncology—”Can We Do Better?”—Lessons Learned Over 50 Years. Neurosurgery.

[3] Seker-Polat F., Pinarbasi Degirmenci N., Solaroglu I., Bagci-Onder T. (2022). Tumor Cell Infiltration into the Brain in Glioblastoma: From Mechanisms to Clinical Perspectives. Cancers.

[4] Haddad A.F., Young J.S., Amara D., Berger M.S., Raleigh D.R., Aghi M.K., Butowski N.A. (2021). Mouse models of glioblastoma for the evaluation of novel therapeutic strategies. Neurooncol. Adv..

[5] Bourré L. Tumor Homograft Model Generation: Dissociated Tumor Cells vs Tumor Fragments—Crown Bioscience. https://blog.crownbio.com/tumor-homograft-models-dtc-tumor-fragments.

[6] Seligman A.M., Shear M.J., Alexander L. (1939). Studies in Carcinogenesis: VIII. Experimental Production of Brain Tumors in Mice with Methylcholanthrene. Am. J. Cancer.

[7] Ausman J.I., Shapiro W.R., Rall D.P. (1970). Studies on the chemotherapy of experimental brain tumors: Development of an experimental model. Cancer Res..

[8] Cha S., Johnson G., Wadghiri Y.Z., Jin O., Babb J., Zagzag D., Turnbull D.H. (2003). Dynamic, contrast-enhanced perfusion MRI in mouse gliomas: Correlation with histopathology. Magn. Reson. Med..

[9] Szatmari T., Lumniczky K., Desaknai S., Trajcevski S., Hidvegi E.J., Hamada H., Safrany G. (2006). Detailed characterization of the mouse glioma 261 tumor model for experimental glioblastoma therapy. Cancer Sci..

[10] Newcomb E.W., Zagzag D., Meir E. (2009). The Murine GL261 Glioma Experimental Model to Assess Novel Brain Tumor Treatments. CNS Cancer. Cancer Drug Discovery and Development.

[11] Delgado-Goni T., Julia-Sape M., Candiota A.P., Pumarola M., Arus C. (2014). Molecular imaging coupled to pattern recognition distinguishes response to temozolomide in preclinical glioblastoma. NMR Biomed..

[12] Candolfi M., Yagiz K., Wibowo M., Ahlzadeh G.E., Puntel M., Ghiasi H., Kamran N., Paran C., Lowenstein P.R., Castro M.G. (2014). Temozolomide does not impair gene therapy-mediated antitumor immunity in syngeneic brain tumor models. Clin. Cancer Res..

[13] El Meskini R., Iacovelli A.J., Kulaga A., Gumprecht M., Martin P.L., Baran M., Householder D.B., Van Dyke T., Weaver Ohler Z. (2015). A preclinical orthotopic model for glioblastoma recapitulates key features of human tumors and demonstrates sensitivity to a combination of MEK and PI3K pathway inhibitors. Dis. Model. Mech..

[14] The Cancer Genome Atlas Research Network (2008). Comprehensive genomic characterization defines human glioblastoma genes and core pathways. Nature.

[15] Dinca E.B., Sarkaria J.N., Schroeder M.A., Carlson B.L., Voicu R., Gupta N., Berger M.S., James C.D. (2007). Bioluminescence monitoring of intracranial glioblastoma xenograft: Response to primary and salvage temozolomide therapy. J. Neurosurg..

[16] Liu S., Su Y., Lin M.Z., Ronald J.A. (2021). Brightening up Biology: Advances in Luciferase Systems for In Vivo Imaging. ACS Chem. Biol..

[17] Schulz J.A., Rodgers L.T., Kryscio R.J., Hartz A.M.S., Bauer B. (2022). Characterization and comparison of human glioblastoma models. BMC Cancer.

[18] Baklaushev V.P., Grinenko N.F., Yusubalieva G.M., Abakumov M.A., Gubskii I.L., Cherepanov S.A., Kashparov I.A., Burenkov M.S., Rabinovich E.Z., Ivanova N.V. (2015). Modeling and integral X-ray, optical, and MRI visualization of multiorgan metastases of orthotopic 4T1 breast carcinoma in BALB/c mice. Bull. Exp. Biol. Med..

[19] Baklaushev V.P., Kilpelainen A., Petkov S., Abakumov M.A., Grinenko N.F., Yusubalieva G.M., Latanova A.A., Gubskiy I.L., Zabozlaev F.G., Starodubova E.S. (2017). Luciferase Expression Allows Bioluminescence Imaging But Imposes Limitations on the Orthotopic Mouse (4T1) Model of Breast Cancer. Sci. Rep..

[20] Sanchez V.E., Lynes J.P., Walbridge S., Wang X., Edwards N.A., Nwankwo A.K., Sur H.P., Dominah G.A., Obungu A., Adamstein N. (2020). GL261 luciferase-expressing cells elicit an anti-tumor immune response: An evaluation of murine glioma models. Sci. Rep..

[21] Enriquez Perez J., Kopecky J., Visse E., Darabi A., Siesjo P. (2020). Convection-enhanced delivery of temozolomide and whole cell tumor immunizations in GL261 and KR158 experimental mouse gliomas. BMC Cancer..

[22] Garbow J.R., Johanns T.M., Ge X., Engelbach J.A., Yuan L., Dahiya S., Tsien C.I., Gao F., Rich K.M., Ackerman J.J.H. (2021). Irradiation-Modulated Murine Brain Microenvironment Enhances GL261-Tumor Growth and Inhibits Anti-PDl1 Immunotherapy. Front. Oncol..

[23] Renner D.N., Malo C.S., Jin F., Parney I.F., Pavelko K.D., Johnson A.J. (2016). Improved Treatment Efficacy of Antiangiogenic Therapy when Combined with Picornavirus Vaccination in the GL261 Glioma Model. Neurotherapeutics.

[24] Carlson B.L., Pokorny J.L., Schroeder M.A., Sarkaria J.N. (2011). Establishment, maintenance and in vitro and in vivo applications of primary human glioblastoma multiforme (GBM) xenograft models for translational biology studies and drug discovery. Curr. Protoc. Pharmacol..

[25] El Meskini R., Atkinson D., Weaver Ohler Z. (2023). Translational Orthotopic Models of Glioblastoma Multiforme. J. Vis. Exp..

[26] Toth L.A. (2000). Defining the Moribound Condition as an Experimental Endpoint for Animal Research. ILAR J..

[27] Wallace J. (2000). Humane endpoints and cancer research. ILAR J..

[28] Cree I.A., Tan P.H., Travis W.D., Wesseling P., Yagi Y., White V.A., Lokuhetty D., Scolyer R.A. (2021). Counting mitoses: SI(ze) matters!. Mod. Pathol..

[29] Detry M.A., Ma Y. (2016). Analyzing Repeated Measurements Using Mixed Models. JAMA.

[30] Burnham K.P., Anderson D.R. (2002). Model Selection and Multimodel Inference: A Practical Information-Theoretic Approach.

[31] Andersen P.K., Gill R.D. (1982). Cox’s Regression Model for Counting Processes: A Large Sample Study. Ann. Stat..

[32] Kalbfleisch J.D., Prentice R.L. (2002). The Statistical Analysis of Failure Time Data.

[33] Huang A. (2017). Mean-parametrized Conway–Maxwell–Poisson regression models for dispersed counts. Stat. Model..

[34] Agresti A. (2002). Categorical Data Analysis.

[35] Searle S.R., Speed F.M., Milliken G.A. (1980). Population marginal means in the linear model: An alternative to least squares means. Am. Stat..

[36] Nakagawa S., Johnson P.C.D., Schielzeth H. (2017). The coefficient of determination R2 and intra-class correlation coefficient from generalized linear mixed-effects models revisited and expanded. J. R. Soc. Interface.

[37] Nagelkerke N.J.D. (1991). A Note on a General Definition of the Coefficient of Determination. Biometrika.

[38] Brooks M.E., Kristensen K., van Benthem K.J., Magnusson A., Berg C.W., Nielsen A., Skaug H.J., Mächler M., Bolker B.M. (2017). glmmTMB balances speed and flexibility among packages for zero-inflated generalized linear mixed modeling. R. J..

[39] Venables W.N., Ripley B.D., Venables W.N. (2002). Modern Applied Statistics with S.

[40] Lenth R.V., Buerkner P., Gine-Vazquez I., Herve M., Jung M., Love J., Miguiz F., Riebl H., Singmann H. emmeans: Estimated Marginal Means, aka Least-Squares Means. https://cran.r-project.org/web/packages/emmeans/.

[41] Lüdecke D., Ben-Shachar M.S., Patil I., Waggoner P., Makowski D. (2021). performance: An R Package for Assessment, Comparison and Testing of Statistical Models. J. Open Source Softw..

[42] Ihaka R., Gentleman R. (1996). R: A Language for Data Analysis and Graphics. J. Comput. Graph. Stat..

[43] Bates D., Mächler M., Bolker B., Walker S. (2015). Fitting linear mixed-effects models using lme4. J. Stat. Softw..

[44] Fayzullin A., Tuvnes F.A., Skjellegrind H.K., Behnan J., Mughal A.A., Langmoen I.A., Vik-Mo E.O. (2016). Time-lapse phenotyping of invasive glioma cells ex vivo reveals subtype-specific movement patterns guided by tumor core signaling. Exp. Cell Res..

[45] McKelvey K.J., Hudson A.L., Prasanna Kumar R., Wilmott J.S., Attrill G.H., Long G.V., Scolyer R.A., Clarke S.J., Wheeler H.R., Diakos C.I. (2020). Temporal and spatial modulation of the tumor and systemic immune response in the murine Gl261 glioma model. PLoS ONE.

[46] Bausart M., Bozzato E., Joudiou N., Koutsoumpou X., Manshian B., Preat V., Gallez B. (2023). Mismatch between Bioluminescence Imaging (BLI) and MRI When Evaluating Glioblastoma Growth: Lessons from a Study Where BLI Suggested “Regression” while MRI Showed “Progression”. Cancers.

[47] Leten C., Struys T., Dresselaers T., Himmelreich U. (2014). In vivo and ex vivo assessment of the blood brain barrier integrity in different glioblastoma animal models. J. Neurooncol..

[48] Yeo A.T., Charest A. (2017). Immune Checkpoint Blockade Biology in Mouse Models of Glioblastoma. J. Cell. Biochem..

[49] Kirschner S., Murle B., Felix M., Arns A., Groden C., Wenz F., Hug A., Glatting G., Kramer M., Giordano F.A. (2016). Imaging of Orthotopic Glioblastoma Xenografts in Mice Using a Clinical CT Scanner: Comparison with Micro-CT and Histology. PLoS ONE.

[50] Bouckaert C., Christiaen E., Verhoeven J., Descamps B., De Meulenaere V., Boon P., Carrette E., Vonck K., Vanhove C., Raedt R. (2021). Comparison of In Vivo and Ex Vivo Magnetic Resonance Imaging in a Rat Model for Glioblastoma-Associated Epilepsy. Diagnostics.

[51] Kober C., Weibel S., Rohn S., Kirscher L., Szalay A.A. (2015). Intratumoral INF-gamma triggers an antiviral state in GL261 tumor cells: A major hurdle to overcome for oncolytic vaccinia virus therapy of cancer. Mol. Ther. Oncolytics.

[52] Ruotsalainen J., Martikainen M., Niittykoski M., Huhtala T., Aaltonen T., Heikkila J., Bell J., Vaha-Koskela M., Hinkkanen A. (2012). Interferon-beta sensitivity of tumor cells correlates with poor response to VA7 virotherapy in mouse glioma models. Mol. Ther..

[53] Thotala D., Karvas R.M., Engelbach J.A., Garbow J.R., Hallahan A.N., DeWees T.A., Laszlo A., Hallahan D.E. (2015). Valproic acid enhances the efficacy of radiation therapy by protecting normal hippocampal neurons and sensitizing malignant glioblastoma cells. Oncotarget.

[54] Sullivan J.K., Fahey P.P., Agho K.E., Hurley S.P., Feng Z., Day R.O., Lim D. (2023). Valproic acid as a radio-sensitizer in glioma: A systematic review and meta-analysis. Neurooncol. Pract..

