# Supplementary material for: Optimization, Characterization, and Comparison of Two Luciferase-Expressing Mouse Glioblastoma Models
Source: Cancers (Basel). 2024 May 24;16(11):1997. doi: 10.3390/cancers16111997 (PMC11171217; doi:10.3390/cancers16111997)
Supplement: Supplementary file 1 [file cancers-16-01997-s001.zip › cancers-2945122-supplementary.pdf]

Figure S1

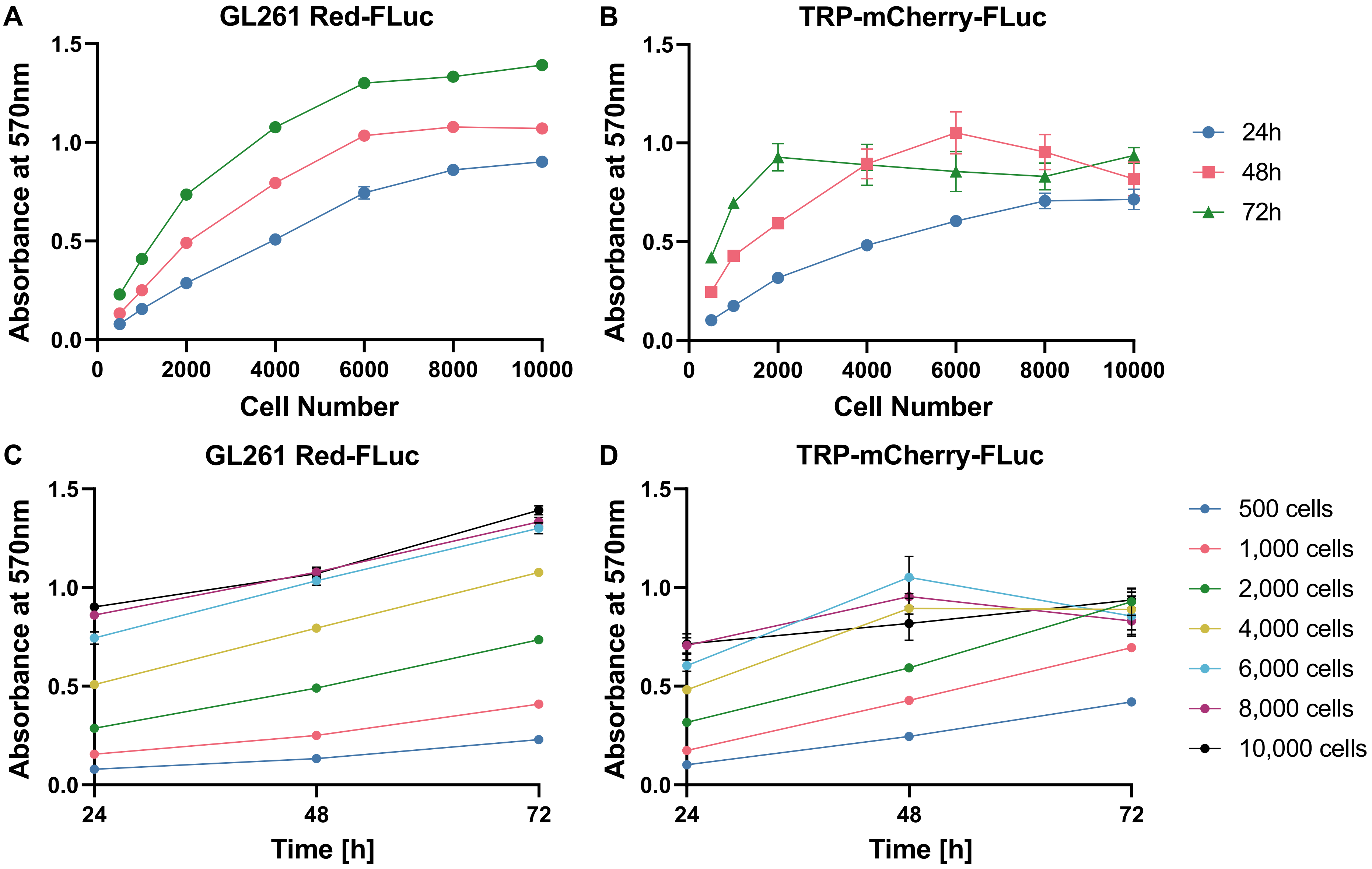

**Effect**	**Estimate**	**χ^2 (df)**	**p**	**R^2**
Time	0.299 ±0.009	1128.908 (1)	< 0.001	0.215
Cells	0.526 ±0.022	561.250 (1)	< 0.001	0.215
GL261	-0.017 ±0.031	0.327 (1)	0.567	0.039
Cells×GL261	0.142 ±0.022	41.204 (1)	< 0.001	0.039
Time×Cells	-0.141 ±0.009	247.725 (1)	< 0.001	0.034
Time×GL261	0.000 ±0.009	0.001 (1)	0.975	0.003
Time×Cells×GL261	0.046 ±0.009	25.968 (1)	< 0.001	0.003

Figure S1. Comparison of GL261 Red-FLuc and TRP-mCF *in vitro* growth characteristics. GL261 Red-FLuc and TRP-mCF cells were loaded into three plates at 0.5, 1, 2, 4, 6, 8, and 10 thousand seeded cells, 6 wells per loading. Cells were tested by MTT assay at 48 and 72 hours. Absorbance at 570 (A570) was compared by generalized linear mixed models with nested random intercepts, specifically well nested within a plate. The fixed-effect model was A570 ~ Glioma line (GL261 vs TRP-mCF) × Time × Cell count, with a log link. The time slopes of the model were estimated by marginal trends. Slopes were used to estimate doubling times by glioma line. A significant three-way interaction among time, cell count, and line was revealed by analysis. As expected, cells proliferated over time, and the initial seed corresponded to higher MTT signals. GL261 had lower A570 readings than TRP-mCF, but this difference was not significant overall. Initial seedings greatly influenced doubling times, with higher seedings corresponding to longer times (**Table S1**). At cell densities higher than 4,000 cells, TRP-mCF cultures reached 100% confluency by 72h.

Figure S2

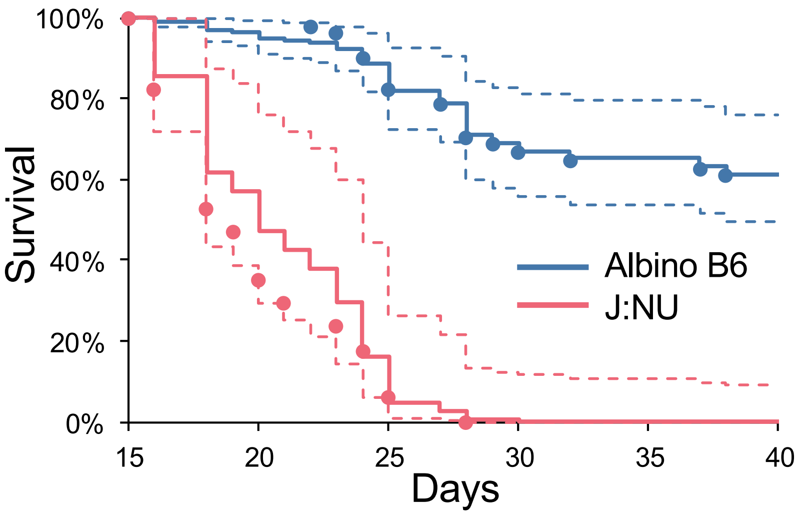

Effect	χ^2 (df)	p	R^2
Mouse Strain	42.87 (1)	< 0.001	0.475

Figure S2. Survival comparison of immunocompetent (albino B6) and immunocompromised (J:NU) mice injected with 50,000 GL261 Red-FLuc cells. Survival modeling (above) and descriptive statistics of modeled data (below). Points represent actual percent survival. Solid lines are Cox model predictions. Dashed lines are 95% CI. (Albino B6: n = 51, J:NU: n = 17).

Table S1

Table S1. *In Vitro* GL261 Red-FLuc and TRP-mCF Growth Characteristics

	Doubling Time [h]	
**Cell Number**	GL261 Red-FLuc	TRP-mCF
**500**	32.9 ±1.6/1.5	26.1 ±1.0/1.0
**1000**	34.1 ±1.6/1.5	27.6 ±1.1/1.0
**2000**	36.7 ±1.6/1.5	31.1 ±1.2/1.1
**4000**	43.3 ±1.8/1.7	41.6 ±1.7/1.6
**6000**	52.9 ±2.9/2.6	62.9 ±4.4/3.8
**8000**	67.8 ±6.6/5.6	129.2 ±27.5/19.3
**10000**	94.4 ±18.7/13.4	NC

[a]Not computed, theoretically negative

Table S2

Table S2. Experiment Summary

Cell Line	Mouse Strain	Cell Number	Median Survival [day post-implantation]	Starting N	Analyzed N*
GL261 Red-FLuc	B6(Cg)-Tyr<c-2J>/J (Jax# 000058)	50,000	27 (accepted tumors)	55	51
GL261 Red-FLuc	J:NU (Jax #007850)	50,000	19	17	17
GL261 Red-FLuc	J:NU (Jax #007850)	25,000	24	10	10
GL261 Red-FLuc	J:NU (Jax #007850)	15,000	24	10	10
GL261 Red-FLuc	J:NU (Jax #007850)	10,000	25	15	15
GL261 Red-FLuc	J:NU (Jax #007850)	5,000	27	15	15
TRP-mCherry-FLuc	B6(Cg)-Tyr<c-2J>/J (Jax# 000058)	50,000	20	5	5
TRP-mCherry-FLuc	B6(Cg)-Tyr<c-2J>/J (Jax# 000058)	30,000	21	5	5
TRP-mCherry-FLuc	B6(Cg)-Tyr<c-2J>/J (Jax# 000058)	20,000	21	5	5
TRP-mCherry-FLuc	B6(Cg)-Tyr<c-2J>/J (Jax# 000058)	10,000	25	5	5
TRP-mCherry-FLuc	B6(Cg)-Tyr<c-2J>/J (Jax# 000058)	5,000	25	5	5

*Four mice excluded due to surgical mortality
